# Caveats in Transneuronal Tracing with Unmodified Rabies Virus: An Evaluation of Aberrant Results Using a Nearly Perfect Tracing Technique

**DOI:** 10.3389/fncir.2016.00046

**Published:** 2016-07-11

**Authors:** Tom J. H. Ruigrok, Sven van Touw, Patrice Coulon

**Affiliations:** ^1^Department of Neuroscience, Erasmus Medical CenterRotterdam, Netherlands; ^2^Equipe P3M – UMR 7298, Institut de Neurosciences de la Timone, Aix-Marseille Université, CNRSMarseille, France

**Keywords:** rabies virus, cerebellum, inferior olive, Purkinje cells, cerebellar nuclei, inferior olivary afferents, neuronal degeneration, viral infection

## Abstract

Apart from the genetically engineered, modified, strains of rabies virus (RABV), unmodified ‘fixed’ virus strains of RABV, such as the ‘French’ subtype of CVS11, are used to examine synaptically connected networks in the brain. This technique has been shown to have all the prerequisite characteristics for ideal tracing as it does not metabolically affect infected neurons within the time span of the experiment, it is transferred transneuronally in one direction only and to all types of neurons presynaptic to the infected neuron, number of transneuronal steps can be precisely controlled by survival time and it is easily detectable with a sensitive technique. Here, using the ‘French’ CVS 11 subtype of RABV in Wistar rats, we show that some of these characteristics may not be as perfect as previously indicated. Using injection of RABV in hind limb muscles, we show that RABV-infected spinal motoneurons may already show lysis 1 or 2 days after infection. Using longer survival times we were able to establish that Purkinje cells may succumb approximately 3 days after infection. In addition, some neurons seem to resist infection, as we noted that the number of RABV-infected inferior olivary neurons did not progress in the same rate as other infected neurons. Furthermore, in our hands, we noted that infection of Purkinje cells did not result in expected transneuronal labeling of cell types that are presynaptic to Purkinje cells such as molecular layer interneurons and granule cells. However, these cell types were readily infected when RABV was injected directly in the cerebellar cortex. Conversely, neurons in the cerebellar nuclei that project to the inferior olive did not take up RABV when this was injected in the inferior olive, whereas these cells could be infected with RABV via a transneuronal route. These results suggest that viral entry from the extracellular space depends on other factors or mechanisms than those used for retrograde transneuronal transfer. We conclude that transneuronal tracing with RABV may result in unexpected results, as not all properties of RABV seem to be ubiquitously valid.

## Introduction

Apart from the genetically engineered modified strains of rabies virus (RABV), unmodified ‘fixed’ virus strains of RABV, such as the ‘French’ subtype of CVS11, are used to examine synaptically connected networks in the brain. Whereas the modified strains are essentially used to study monosynaptic connections (Callaway and Luo, 2015), CVS11 is predominantly used to study oligosynaptic connections (Ugolini, 2010). In 1995, the first detailed report using RABV as a transneuronal tracer demonstrated the advantages of this technique over other viral tracing techniques in identifying synaptically connected neuronal circuits within the brain (Ugolini, 1995). Subsequent studies have elaborated the superiority of rabies transneuronal tracing in many different systems (e.g., Tang et al., 1999; Kelly and Strick, 2000, 2003; Ugolini, 2010; Lu et al., 2012). Indeed, RABV has been used successfully as a transneuronal tracer in mice (Bras et al., 2008; Coulon et al., 2011; Kasumacic et al., 2015), rats (Ugolini, 1995; Tang et al., 1999; Morcuende et al., 2002; Ruigrok et al., 2008; Salin et al., 2008), guinea pig (Graf et al., 2002), and monkey (Kelly and Strick, 2003; Moschovakis et al., 2004; Prevosto et al., 2010).

In these and other experimental designs (Tang et al., 1999; Salin et al., 2008; Suzuki et al., 2012) transneuronal labeling with rabies has not failed to label known pathways mediated by classical chemical synapses (Ugolini, 2010). The only exceptions were reported for the locus coeruleus which was not or only poorly infected in several early studies (Astic et al., 1993; Ugolini, 1995), and nitrergic interneurons of the striatum, which were not labeled as second-order neurons after RABV injection in basal ganglia output structures (Salin et al., 2009). This phenomenon may be attributed as a characteristic of catecholaminergic modulatory systems that use ‘volume transmission’ rather than ‘wiring transmission’ (Fuxe et al., 2007; Ugolini, 2010). Indeed, the use of rabies has revealed many new connections that, by using classic retrograde and anterograde labeling, have escaped detection (e.g., Bostan et al., 2010; Levinthal and Strick, 2012).

In her comprehensive methods paper on transneuronal viral tracing, Ugolini (2010; also see Ugolini, 2011) has listed several characteristics that would be vital for a substance to be classified as an excellent transneuronal tracer. The tracer should (1) only be exchanged by synaptically connected neurons; (2) only allow transneuronal transfer within one direction; (3) allow easy identification of the number of synaptic transfers (order of labeling); (4) label all groups of higher-order neurons that are synaptically connected to the transferring neurons; (5) be easy to detect and not disappear over time and (6) not alter neuronal metabolism. Ugolini concludes that the ‘French’ subtype of CVS11 essentially fulfills all these prerequisites and represents a perfect tracer to study linked functional circuitries in the brain.

In the Wistar rat, we have used the same CVS11 strain of RABV that was also used by Ugolini and collaborators in several neuroanatomical studies (Ruigrok et al., 2008; Suzuki et al., 2012). However, in these and in subsequent studies, we have noted that this strain of RABV does not always satisfy the conditions listed above. Here, we like to specifically report on some potential pitfalls that should be taken into account when using the CVS11 strain of RABV as a retrograde transneuronal tracer. From our studies we conclude that (1) some areas with infected cells are prone to show gliosis at an early stage and neurons may even degenerate within a few days after infection; (2) some types of cells may show resilience to infection or (3) may fail to become infected within the expected time interval. We like to stress that this study only reports on genetically unmodified strains of RABV.

## Materials and Methods

This study elaborates on data obtained during several earlier studies (Ruigrok et al., 2008; Suzuki et al., 2012) that were centered on different research questions. In addition, it contains results from a new and as yet unpublished dataset.

### Surgical Procedures and Injections

**FIGURE 1:**
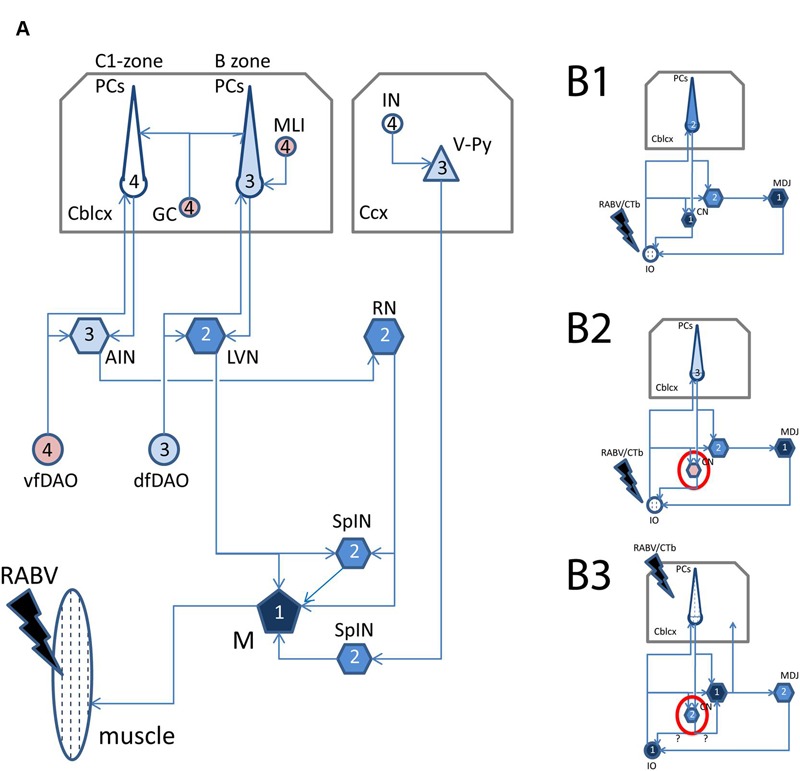
**Schematized diagram of relevant circuitry after RABV injections in muscles, inferior olive, or cerebellar cortex. (A)** Relevant supraspinal connections to spinal motoneurons. Note that arrows indicate direction of information transfer and that transfection of RABV is in the opposite (retrograde) direction. After injection of RABV in skeletal muscles (hatched), first-order labeling is observed in motoneurons, followed by second-order labeling in SpIN, RN, LVN, third-order labeling in Purkinje cells of the B-zone as well as of neurons in the cerebellar nuclei that project to the RN, olivary neurons that provide collaterals to the LVN and layer V pyramidal cells in the somatosensory cortex of the cerebrum. Finally, fourth-order labeling is represented by Purkinje cells in the C1-zone, ventral fold of the DAO, molecular layer interneurons of the cerebellar cortex and interneurons of the cerebral cortex. **(B1)** Relevant connections after injection of RABV/CTb in the inferior olive (hatched). First-order labeling is expected at the MDJ and of nucleo-olivary neurons in the cerebellar nuclei, followed by second-order labeling of PCs that terminate on infected nucleo-olivary neurons, and third-order labeling of Purkinje cells that selectively target cerebellar nuclear neurons presynaptic to infected MDJ neurons. **(B2)** Same situation shown in **(B1)**, however, now with failure of nucleo-olivary infection. **(B3)** Relevant connections after RABV/CTb injection in the cerebellar cortex. In this situation first-order labeling is expected in olivary neurons and nucleocortical neurons. Second-order labeling of nucleo-olivary neurons could occur by way of infected olivary neurons or infected nucleo-cortical neurons. See Results, section on *“Small Neurons in the Cerebellar Nuclei Can Be Infected Transneuronally”* for further information. Black filling indicates first-order infection, dark blue filling indicates second-order infection, light blue filling indicates third-order infection, no filling indicates expected fourth-order infection. Pink filling indicates poor or failure of infection. Red circle in **(B2,B3)** indicates contrasting labeling of nucleo-olivary neurons in both injection situations. Midline crossing of connections is not indicated. Abbreviations: AIN, anterior interposed nucleus; Cblcx, cerebellar cortex; Ccx, cerebral cortex; CN, cerebellar nuclei; dfDAO, dorsal fold of dorsal accessory olive; GC, granule cells; IN, cerebral cortical interneuron; IO, inferior olive; LVN, lateral vestibular nucleus; M, motoneurons; MDJ, mesodiencephalic junction; MLI, molecular layer interneurons; PCs, Purkinje cells; RN, red nucleus; SpIN, spinal interneurons; vf-DAO, ventral fold of dorsal accessory olive; V-Py, layer V pyramidal cells.

All experiments were carried out using adult male Wistar rats (200–300 g). Surgical procedures adhered to the European guidelines for the care and use of laboratory animals and were approved by the ethics committee in Neurosciences at the Institut de Neurosciences de la Timone, INT-Marseille (n° 02167-01). Vaccinated personnel conducted all RABV handling, surgery and animal care procedures at the appropriate biosafety containment level (level 2) in the lab of PC (Marseille). In all cases but one the unmodified ‘French’ fixed strain of the Challenge Virus Standard (CVS) 11 was used as a tracer. In the remaining case the N2c strain of CVS 24 was injected. For a review on the different CVS strains, see Ugolini (2010). The present study reports on RABV data from three types of experiments: i.e., RABV injection in muscles, RABV injection in the inferior olive and RABV injection in the cerebellar cortex (**Table 1**). The relevant circuitry that will be analyzed in the results is schematized in **Figures 1A,B**. In all experiments, the rats were terminated before onset of obvious disease signs.

**Table 1:** List of experiments described in the present study.

Injection target	*N*	Injectate	pfu	Volume	Survival	Reference
Muscle	20 (25)^∗^	CVS11	1–2×10^7^	5–30 μl	78–192 h	Ruigrok et al., 2008
Cerebel. cortex	3 (18)^∗^	CVS11 + CTbˆ	4800	150 nl	48–50 h	Suzuki et al., 2012
Inferior olive	10	CVS11 + CTbˆ	4800	150 nl	30–68 h	–
Inferior olive	1	CVS-N2C + CTbˆ	4800	150 nl	48 h	–
Mesodien. Junct.	3	CTb (1%)	–	Iontophoresis	120 h	–

*^∗^N, number re-analyzed for this study, number between brackets refers to total number of cases used in the referenced study.*

*ˆFinal concentration of CTb in injectate was 0.2% w/v.*

#### RABV Injection in Muscles

Technical data on RABV injection in either hind limb or forelimb muscles were published in Ruigrok et al. (2008). This set of data comprises 25 cases with RABV injections in either anterior tibial, gastrocnemius, flexor digitorum, and extensor digitorum muscles and using survival times ranging from 3 to 8 days. Briefly, under Ketamine/Xylazine anesthesia, the skin overlying the muscles was opened and under direct visual guidance muscles were injected in multiple penetration tracks with a total of 10–30 μl RABV (1 or 2 × 10^7^ plaque forming units (PFU)/rat, volume depending upon size of muscle). In most cases the used titer and volume was sufficient to result in robust infection of the central nervous system (Ruigrok et al., 2008). After suturing the skin the animals were allowed to recover and were housed in pairs in standard cages within a hooded and air-filtered cabinet within the lab at biosafety containment level 2.

#### RABV Injection in the Inferior Olive

The inferior olive was injected in 11 rats. Animals were anesthetized intraperitoneally with a mixture of ketamine (60 mg/kg: Imalgene, Merial) and xylazine (10 mg/kg, Rompun, Bayer) and was supplemented when required. The animals were subsequently placed in a stereotactic frame (David Kopf Instruments) according to the atlas by Paxinos and Watson (1998). The skin overlying the occipital bone and dorsal neck muscles was cut, the neck muscles were separated and the foramen magnum was slightly enlarged by removing the lower rim of the occipital bone. In this way, after careful lengthwise opening of the occipito-atlantal membrane and dura, the lower medulla and upper spinal cord became visible. Injections were made with an adapted Hamilton syringe of 10 μl, the plunger of which was moved by a programmable microsyringe pump (flow: 200 nl/min) and which was connected by way of thin tubing (inner diameter 0.1 mm) in which the movement of an air bubble was monitored to enable further control of the injected volume. The tubing was subsequently connected to a thin isolated Hamilton needle containing the injectate.

The injectate consisted of a mixture of one part cholera toxin, b-subunit (CTb: low salt: List Biological Laboratories, 1% w/v in 0.2 M phosphate buffer, pH 7.4) and four parts RABV. RABV consisted of a cell culture supernatant in minimal essential medium titrating 4 × 10^7^ PFU/ml. One rat was injected with the N2c strain, all others with the ‘French’ CVS11 strain (see above). A total of approximately 150 nl was injected at: AP, obex level; laterality, 0.3–0.4; depth, 2.8–3.0 mm with a forward penetration angle of 45° in the sagittal plane (Ruigrok and Voogd, 2000). After injection the needle was left *in situ* for 5 min. These parameters without exception resulted in a spread of the injectate [as visualized by CTb immunohistochemistry (see Prevosto et al., 2010; Suzuki et al., 2012)] that was centered on the olivary complex covering approximately half of its volume without obvious invasion of the overlying reticular formation. After injection the neck muscles were sutured in layers, the skin was clamped and the animal was allowed to recover. Survival times were chosen at 30 h (*n* = 1); 48–50 h (*n* = 6) or 66–68 h (*n* = 4). The rat receiving the N2c injection survived for 48 h.

#### RABV Injection in the Cerebellar Cortex

Technical data on RABV injection of the cerebellar cortex have been published by Suzuki et al. (2012). Here, we report on new data from three rats with an injection in the intermediate part of the paramedian lobule (PML) and that belonged to the set of experiments further detailed in that paper. Briefly, using a similar approach as used for the inferior olive injections (see above) but with a somewhat larger opening of the occipital bone overlying the caudal cerebellum, visual observation of the PML was obtained. Using a horizontal approach, 150 nl of the same CTb/RABV mixture used for the inferior olive injections, was made in the intermediate PML at a depth of 0.4–0.5 mm below the cerebellar surface. After closure of the wound these three animals survived for 48–50 h.

#### CTb Injection at the Mesodiencephalic Junction

Technical data on CTb injections of the mesodiencephalic junctions (MDJs) have been reported by Ruigrok and Teune (2014). Briefly, in three rats, anesthetized with a cocktail of ketamine and xylazine and mounted in a stereotactic frame, a hole was drilled in parietal bone and, using a glass micropipette with a tip of 10–15 μm, an iontophoretic (4 μA anodal, 7 s on, 7 s off for 10 min) injection with CTb (1% in PB) was made at coordinates: AP +5, lateral 0.8, depth 6.5 mm. After injection, wounds were sutured, and the animal survived for 4 or 5 days.

### Histology

Histological procedures were similar to those described earlier (Ruigrok et al., 2008; Suzuki et al., 2012; Ruigrok and Teune, 2014) and will only be briefly described here. After the appropriate survival time, animals were deeply anesthetized with an overdose pentobarbitone (100 mg/kg i.p.: Nembutal, CEVA, Santé Animale), the thorax was opened and the animal was transcardially perfused with an initial rinse of 0.9% saline (200 ml) followed by 300–400 ml of freshly prepared 4% paraformaldehyde in phosphate buffer with saline (0.9%: PBS). Brain and spinal cord were subsequently removed, post-fixed for several days to completely inactivate the virus (Kelly and Strick, 2000), rinsed overnight in PB with 10% sucrose and embedded in gelatin (11% in 30% sucrose), which was hardened in 10% formalin with 30% sucrose for 3 h. After an overnight rinse in PB with 30% sucrose, the gelatin blocks were cut coronally at 40 μm with a freezing microtome (Leica SM2000R). Sections were collected serially in eight numbered glass vials.

Selected vials were incubated for 48–72 h at 4°C in anti-RABV phosphoprotein mouse monoclonal antibody diluted 1:5000 in PBS with 2% normal horse serum and 0.5% Triton X-100 (PBS+). This antibody (31G10) was isolated in the laboratory Virologie Moléculaire et Structurale at Gif-sur-Yvette, France (Raux et al., 1997), and has been successfully used in many tracing studies (Ruigrok et al., 2008; Salin et al., 2008; Coulon et al., 2011; Suzuki et al., 2012; Kasumacic et al., 2015). After rinsing, sections were incubated in rabbit anti-mouse horseradish peroxidase (90 min, 1:200, Dako), rinsed and finally, to visualize RABV-infected cells for light microscopy (LM), incubated with DAB (0.025% DAB and 0.005% H_2_O_2_ in PB). Other vials were incubated for CTb-immunohistochemistry (Ruigrok and Apps, 2007) using incubation for 48–72 h (4°C) in goat anti-CTb (List Biological Laboratories, diluted 1:15.000 in PBS+). After subsequent incubation in biotinylated rabbit anti-goat (Vector Laboratories) for 90 min, rinsing, they were processed for DAB visualization. All sections processed for LM were mounted serially from chrome alum solution, dried, counterstained with thionin, dehydrated in alcohol series, cleared in xylene and coverslipped with Permount.

Some vials, selected for dual fluorescent immunolabeling of RABV and CTb were incubated for 48–72 h (4°C) with a mixture of the monoclonal anti-RABV and goat anti-CTb in PBS+. After rinsing sections were incubated for 2 h with the secondary antibodies donkey anti-goat-Cy3, 1:200 and donkey anti-mouse-FITC, 1:200 in PBS+ (Jackson ImmunoResearch Europe, Inc.). Other vials, in order to examine double labeling of RABV and choline acetyl transferase (ChAT), were heated for 30 min at 80°C in 25 mM sodium citrate (pH8.75) in order to improve antigen retrieval (Ezaki, 2000), incubated in a mixture of mouse anti-RABV phosphoprotein (Raux et al., 1997) and goat anti-ChAT (1:500, Chemicon) for 48–72 h (4°C) in PBS+. Sections for fluorescent microscopy (FM) were also mounted serially from chromic alum, coverslipped with Vectashield HardSet Mounting Medium (H-400, Vector Laboratories) and stored in the dark in dust free boxes at 4°C.

Immunohistochemical procedures of animals with CTb injection only was similar to that described for CTb above.

### Analysis

Sections for LM were examined with a Leica DMR microscope equipped with a digital camera (Leica DFC-450). Photopanels were constructed with Adobe Photoshop and Illustrator CS6. The 3D-plots of the spinal cords of **Figures 3D1–3** were made using a motorized Olympus BH microscope equipped with a Lucivid miniature monitor and Neurolucida^TM^ software (Microbrightfield). Cells were determined to be motoneurons if they were located within the lateral motoneurons column, had a diameter of at least 25 μm. Cells were only plotted if they contained a nucleus. RABV deposits reflecting lytic motoneurons were plotted if the size of the deposit was at least 40 μm. The Neurolucida set-up was also used to count and measure contours of labeled neurons. Diagrams were constructed with Excel^TM^ (Microsoft Office 2010).

Fluorescent labeling was assessed using either a Leica DMRBE microscope and appropriate filters and photographed with a Hamamatsu camera (C4880) or with a Zeiss LSM700 confocal microscope and Zeiss 2009 software (Zen^TM^). Photopanels were constructed in Adobe Photoshop and Illustrator CS6.

## Results

Progressive RABV infection of neurons within the central nervous system was followed by immunohistochemical labeling using a mouse monoclonal antibody directed against RABV phosphoprotein. This antibody can readily recognize RABV-infected neurons as initially it resulted in labeling of many round cytoplasmic inclusions, corresponding to Negri bodies (Miyamoto and Matsumoto, 1967; Ni et al., 1996). In addition, after more prolonged infection, the antibody also provided a somewhat opaque to intense dark, even, labeling of the cytoplasm that also invaded the distal dendrites. At that time the proximal part of the axon could be labeled as well. Frequently, however, other changes were noted in and around the infected neurons that have not been specifically documented before and will be highlighted below.

### Invasion of Microglia

**FIGURE 2:**
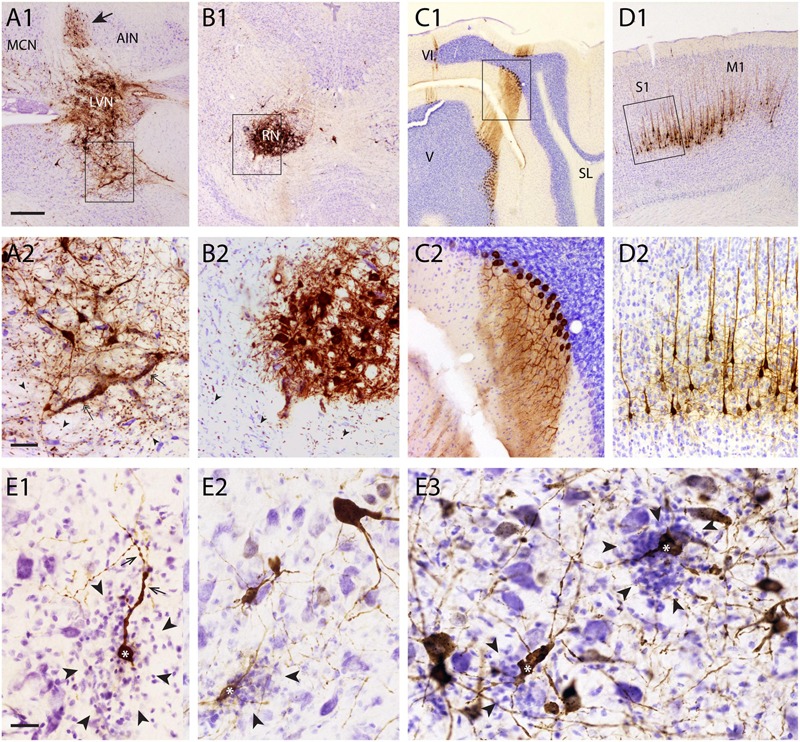
**Gliosis of areas with high density of RABV-infected neurons. (A1,2–D1,2)** examples showing overview **(A1–D1)** and detail **(A2–D2)** of RABV labeling resulting from RABV injection of the right anterior tibial muscle (survival time 5 days) in the ipsilateral LVN **(A1,A2)**, contralateral RN **(B1,B2)**, Purkinje cells of the ipsilateral B-zone **(C1,C2)**, and contralateral sensorimotor cortex (S1, M1). RABV labeling of the Purkinje cells and layer V pyramidal cells are final stages at this survival time. No gliosis is evident in these areas. LVN and RN are presynaptic to RABV-labeled Purkinje cells of the B-zone and RABV-infected neurons (arrow in **A1**) in the anterior interposed nucleus (AIN), respectively. Note that within and around the infected LVN and RN the density of small microglia-like profiles has clearly increased (arrowheads). Rectangles in **(A1–D1**) are enlarged in **(A2–D2)**. **(E1–3**), gliosis (arrowheads) around RABV-infected neurons (marked with ^∗^) in the cerebellar nuclei 48 h after injection of the cerebellar cortex. Dendritic appearance of RABV-infected neurons surrounded by microglia was frequently irregular and beaded (arrows). Note that no obvious gliosis was present around many other RABV-labeled neurons. Abbreviations: LVN, lateral vestibular nucleus; MCN, medial cerebellar nucleus; RN, red nucleus; V, VI, lobules V, VI; SL, simple lobule. Scale bars represent 500 μm for **(A1–D1)**, 100 μm for **(A2–D2)**, 25 μm for **(E1–3)**.

Areas with dense accumulation of RABV-infected neurons frequently also showed an enhanced invasion of microglia. **Figures 2A1–D2** shows RABV infection from a case where the animal was sacrificed 5 days after RABV injection in the anterior tibial muscle. At this stage, the transneuronal labeling had progressed to infection of layer 5 pyramidal neurons in the contralateral sensorimotor cortex and of Purkinje cells in the ipsilateral cerebellar cortex (i.e., third-order labeling, cf **Figure 1A**). No transneuronal labeling beyond both cell types was noted (i.e., of interneurons in either the cerebral or cerebellar cortices, respectively: **Figures 2C2–D2**). In the same animal prominent labeling of two premotor structures, the contralateral red nucleus (RN) and the ipsilateral lateral vestibular nucleus (LVN), was also evident. Although in both structures gliosis was apparent (**Figures 2A2,B2**), it was more pronounced in the LVN. Both structures were unmistakably labeled as the second to last step in the transneuronal transport chain. The infected Purkinje cells were located in the lateral vermis, where they occupied the B-zone, which is presynaptic to the LVN (Voogd and Ruigrok, 2004; Ruigrok et al., 2008), whereas the medial part of the anterior interposed nucleus (arrow in **Figure 2A1**) is presynaptic to the ventrolateral half of the magnocellular RN (Ruigrok, 2004; Ruigrok et al., 2008). Purkinje cells that target this region of the AIN are located in the paravermis, which, at this stage of infection, did not contain any RABV-labeled Purkinje cells.

Although gliosis was most prominent in cases where fairly large quantities of virus (10–30 μl) had been injected into muscles, it was, more occasionally, also observed after injection of small quantities (150–200 nl) into the CNS itself. Several examples are shown in **Figures 2E1–3** where, in an experiment where RABV was injected in the cerebellar cortex 48 h earlier [sufficient for second-order labeling (Suzuki et al., 2012), also see **Figure 1B3** for relevant connections], a mixture of first- and second-order infected neurons were labeled in the cerebellar nuclei. Note that the dendrites of several RABV-infected neurons were surrounded by microglia and often showed an irregular and beaded appearance. No obvious malformations were seen of infected neurons that were not heavily surrounded by microglia.

### Degeneration of Infected Neurons

**FIGURE 3:**
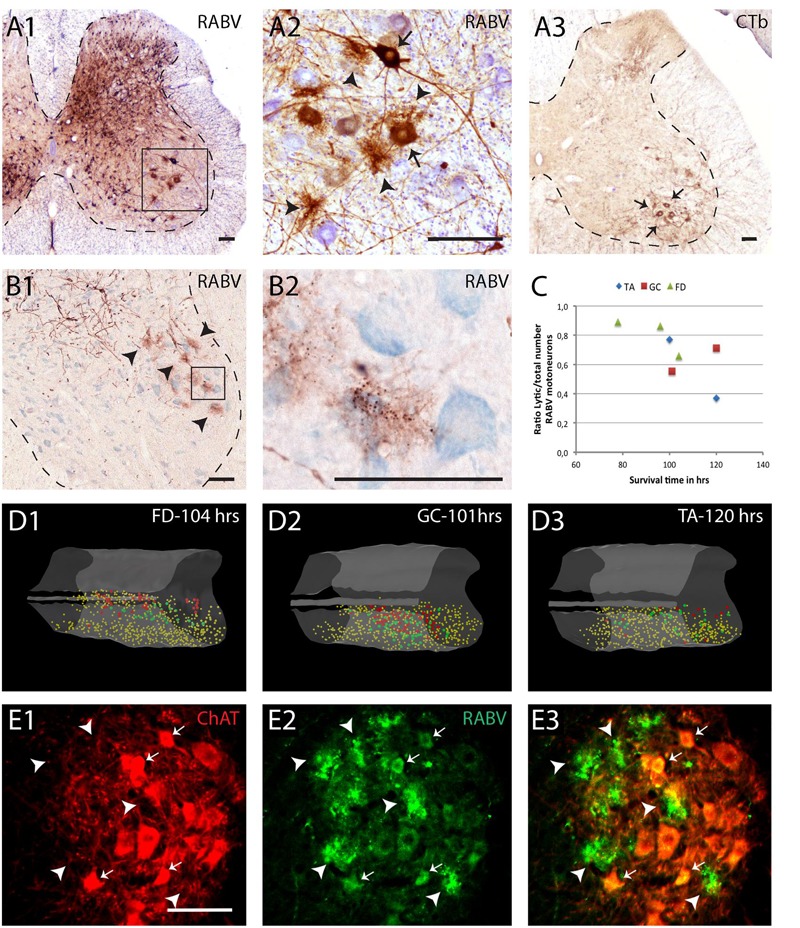
**Early degeneration of RABV-infected spinal motoneurons. (A1,2)**, RABV-infected motoneurons and interneurons in the spinal cord 5 days after injection of the gastrocnemius muscle. Box in **(A1)** is enlarged in **(A2)**. Note, apart from abundant gliosis RABV-labeled motoneurons (arrows) in lamina IX (ventral horn). Intermingled and surrounding these neurons are several deposits with the general size of motoneurons but without a clear boundary. **(A3)** Location of retrogradely labeled neurons after CTb injection of the gastrocnemius muscle. Note that the approximate position of the labeled neurons is similar to the population of RABV-labeled large neurons in lamina IX **(A1)**. **(B1,2)**, examples of similar deposits of reaction product after RABV injection of the flexor digitorum muscle 78 h earlier. Arrowheads in **(B1)** point to presumed degenerated RABV-labeled motoneurons. Boxed area is enlarged in **(B2)** showing that the concentrations of reaction product contain the immunoreactive inclusions that are characteristic of RABV-infected neurons (Kelly and Strick, 2000). **(C)**, graph depicting the ratio of lytic RABV deposits and the total number of labeled large neurons (i.e., sum of lytic deposits and normal RABV labeled somata) within the lateral motoneuron column of the ventral horn in seven analyzed cases. FD, flexor digitorum muscle; GC, gastrocnemius muscle; TA, tibialis anterior muscle. **(D1–3)** 3D reconstructions (based on 28 consecutively plotted sections from a one out of four series) of part of the cervical **(D1)** or lumbosacral **(D2,3)** intumescence in which the location of large neurons presumed to be motoneurons of the lateral motoneuron column are indicated in yellow, in large red dots the location of the lytic RABV deposits and in green normal presumed RABV infected motoneuronal somata. Injections were made in the flexor digitorum (FD: **D1**), gastrocnemius (GC: **D2**) and tibialis anterior (TA: **D3**) muscles. Note that in all three cases the red and green dots are completely intermingled. **(E1–3)**, Double immunofluorescence showing ChAT immunoreactivity (red) in a motoneuron pool of the same animal shown in **(B1,2)** and RABV immunoreactivity in green. Arrowheads point to RABV deposit clusters that were all ChAT-negative. Only some profiles with intact membranes were double labeled (arrows). Scale bars represent 100 μm.

In our material, we have noted that most cell types retain their normal morphological appearance after infection with RABV. However, it was also observed that specific cell types seem to suffer from RABV infection. **Figure 3** shows that selected motoneurons, 5 days after RABV injection into the gastrocnemius, are clearly infected as are many interneurons throughout the ipsilateral and contralateral half of the spinal cord (**Figure 3A1**). However, the groups of clustered and well-labeled motoneurons invariably contained deposits of DAB reaction product that were not confined within sharp neuronal contours but were spread-out (**Figures 3A2,B1**). Yet, within these deposits dark, round inclusions were observed that resembled the cytoplasmic inclusions (Negri bodies) found in other RABV-infected neurons (**Figure 3B2**). The deposits were not observed outside of the clustered group of RABV-labeled motoneurons (**Figures 3D1–3**). Indeed, double labeling with ChAT revealed that although the deposits themselves were ChAT-negative, they otherwise adhered to the clustered location of motoneurons (**Figures 3E1–3**). We therefore suggest that these deposits of reaction product reflect RABV-infected motoneurons that had disintegrated due to a lytic process. Similar deposits, located within motoneuronal columns were observed in 11 out of 13 cases with a RABV injection into either the gastrocnemius or tibialis anterior muscle and a survival time ranging from 3 to 5 days (Ruigrok et al., 2008). Since the earliest infected motoneurons after muscle or nerve injection in rodents are seen 2 days after injection (Tang et al., 1999; Graf et al., 2002; Morcuende et al., 2002; Ruigrok et al., 2008), we conclude that this process of motoneuronal cell degeneration may already start 1 or 2 days after infection. A quantitative assessment of the RABV labeled lytic profiles and motoneuronal profiles performed in seven cases indicates that on average 69% + 6.4 (SEM) of the presumed labeled motoneurons in any case shows lysis. There is a tendency that shorter survival times correlate with a higher ratio of lytic to non-lytic motoneurons (**Figure 3C**), suggesting that lysis may occur already early after infection and that lytic debris, over time, is removed from the neuropil.

**FIGURE 4:**
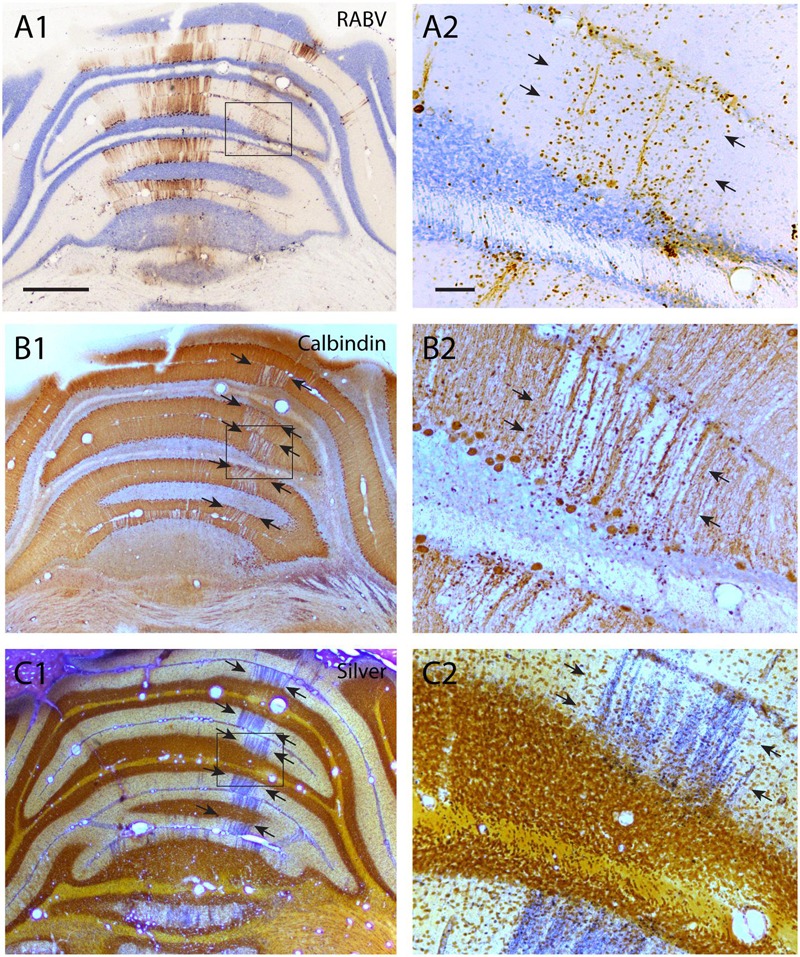
**Degeneration of RABV-infected Purkinje cells. (A1,2)** RABV-infected Purkinje cells in vermis and paravermis 8 days after injection of the right biceps femoris muscle. Boxed area in **(A1)** is enlarged in **(A2)** and indicates gliosis and the disappearance of RABV-infected Purkinje cells of the B-zone (arrows, cf **Figure 2C1**). **(B1,2)** Adjacent sections immunostained for calbindin show a gap in calbindin labeling at the location of the B-zone (between arrows). Boxed area in **(B1)** is enlarged in **(B2)**. **(C1,2)** Agyrophilic staining for neuronal degeneration of an adjacent section indicates severe degeneration of Purkinje cells within the B-zone. Scale bars equal 1000 μm for **(A1–C1)** and 100 μm for **(A2–C2)**.

In another experiment, it was noted that the vulnerability of different cell types is not similar. **Figure 4** shows data from a case with an RABV injection into the biceps femoris muscle and a survival time of 8 days. As this represented an overly long incubation time, the infection had progressed to show considerable bilateral infection of the cerebral cortex, also involving thalamus and the basal ganglia. In the cerebellum prominent labeling was present in the cerebellar nuclei (not shown) as well as of the cerebellar cortex, where many Purkinje cells were labeled at both sides of the brain. However, the strip of early-infected Purkinje cells making up the B-zone ipsilateral to the injection was not apparent (**Figures 4A1,2**). Indeed, many of the Purkinje cells of the B-zone appeared missing after labeling with anti-calbindin (**Figures 4B1,2**) and instead this zone showed prominent degeneration as indicated by silver deposits after treating sections with an agyrophilic degeneration procedure (**Figures 4C1,2**) (Haasdijk et al., 2002). No neuronal degeneration was observed in other nuclei, such as the RN and the LVN that had been infected before the Purkinje cells of the B-zone. As, after injection of hind limb muscles, the earliest labeling of Purkinje cells was reliably observed after 5 days, when infection started in the B-zone (Ruigrok et al., 2008), we conclude that Purkinje cells may already succumb to RABV infection approximately 3 days after infection.

### Reluctant or Absence of RABV Infection

#### Inferior Olivary Cells Show Resilience against Infection

**FIGURE 5:**
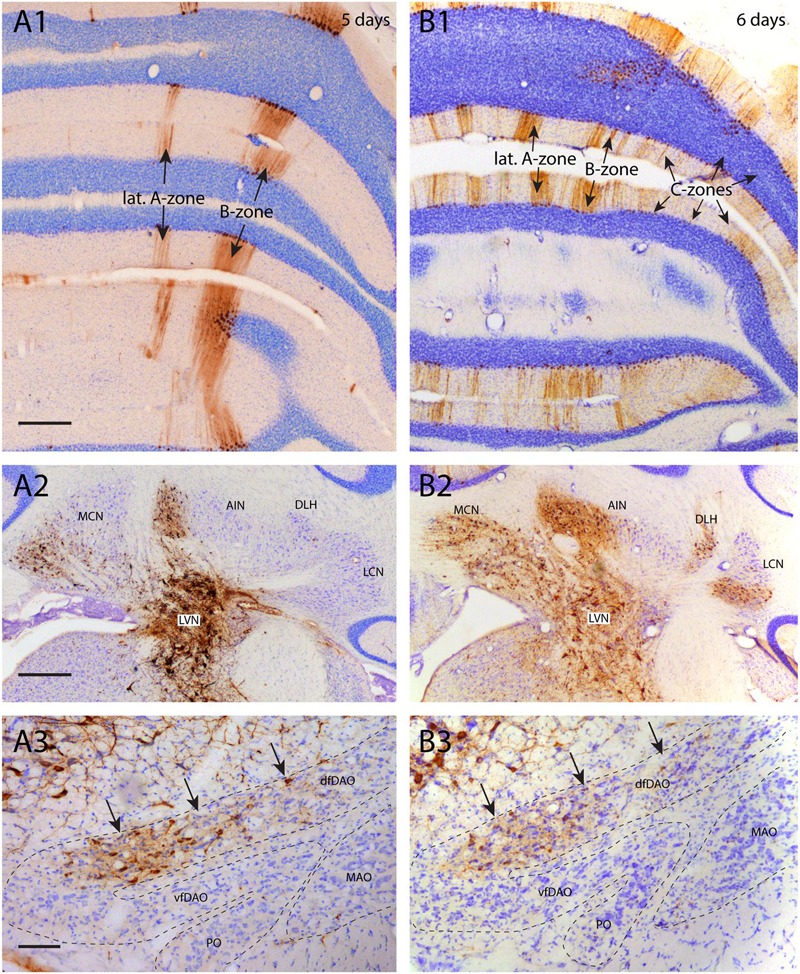
**Poor infection progress of inferior olivary neurons. (A1–3)** RABV infection after injection of the right anterior tibial muscle 5 days before. **(A1)** At this time the first infection of Purkinje cells was apparent, forming a 200–300 μm wide strip of cells that was identified as the B-zone. A more narrow (50–100 μm) medially located band of Purkinje cells was also infected. No other RABV^+^ Purkinje cells were present. The Purkinje cells of the B-zone are known to target the LVN which was heavily labeled **(A2)**. **(A3)** RABV-infected neurons in the inferior olive. Neurons were only located in the caudolateral part of the MAO (not shown) and in the dfDAO (arrows). This latter part of the olive is known to provide climbing fibers to the B-zone and collaterals to the LVN. Therefore, they are expected to belong to the same order of labeling. However, the number of RABV-infected olivary neurons (*n* = 115 counted in one out of every four sections) does not seem to reflect the number of infected Purkinje cells (*n* = 1592 counted in one out of every four sections of the anterior lobe only). **(B1–3)** RABV infection of the same areas seen 6 days after injecting the anterior tibial muscle thereby allowing for an additional transneuronal step. **(B1)** Apart from labeling of the B-zone many additional zones in vermis and hemisphere are infected. This can be clearly related to the infection of the cerebellar nuclei (MCN and AIN) shown in **(A2)**. **(B2)** Expansion of infected areas of the cerebellar nuclei was also noted. **(B3)** Strikingly, despite the exponential increase of the number of infected Purkinje cells (*n* = 7458, counted in a one out of four series of sections of the anterior lobe only, the number of infected olivary cells (arrows, *n* = 139, one out four series of sections) was comparable to the situation in **(A3)**. Scale bars represent 500 μm **(A1,B1,A2,B2)** and 100 μm **(A3,B3)**. Abbreviations: dfDAO, dorsal fold of dorsal accessory olive; DLH, dorsolateral hump; LCN, lateral cerebellar nucleus, LVN, lateral vestibular nucleus; MCN, medial cerebellar nucleus, PO, principal olive; vfDAO, ventral fold of dorsal accessory olive.

In some experimental designs it was observed that the progress of RABV infection was hampered for particular cell types. As an example we examined the infection of inferior olivary neurons as the result of RABV injection in hind limb muscles (also see Ruigrok et al., 2008). **Figure 5** illustrates two cases, both with an injection in the tibialis anterior muscle but with a survival time of 5 days (**Figures 5A1–3**) and 6 days (**Figures 5B1–3**), respectively. In the first case (5 days), infection was profuse in the LVN but also incorporated the medial cerebellar nucleus and the anterior interposed nucleus. LVN infection already had progressed to the next station as labeling was observed in Purkinje cells of the lateral A- and B-zones of the anterior lobe vermis (**Figure 5A1**), which are known to target the LVN (Voogd and Ruigrok, 2004). Note that in this case no Purkinje cell labeling was found that could be presynaptic to the labeling of the anterior interposed nucleus (**Figures 5A1,2**). This case also showed infection of neurons within the dorsal fold of the dorsal accessory olive (dfDAO; **Figure 5A3**), which would be expected because these olivary neurons, known to provide climbing fibers to the B-zone, also are known to collateralize to the LVN (Ruigrok and Voogd, 2000). However, at this time, also as expected, no labeling to the ventral DAO fold, being presynaptic to the anterior interposed nucleus, was noted (**Figure 5A3**, cf **Figure 1A**). However, the second case, allowing 1 day extra for progression of the infection, not only labeled additional regions of the cerebellar nuclei (e.g., lateral cerebellar nucleus and dorsolateral hump; **Figure 5B2**) but also demonstrated a more abundant infection of areas already containing infected neurons 5 days after injection. This occurred simultaneously with a profound elaboration of the infection of Purkinje cells, incorporating the C-zones of the paravermis (**Figure 5B1**). Yet, the striking exception here was the lack in progression of the infection within the inferior olive as this was, both in distribution as well as in number of labeled cells, virtually similar to the labeling observed after 5 days (**Figure 5B3**). This suggests that the olivary neurons that are presynaptic to, e.g., the medial part of the anterior interposed nucleus (which was already infected after 5 days; **Figure 5A2**), were not infected after 6 days (cf. **Figure 1A**). Indeed, counts of infected neurons showed that despite an almost fivefold increase in the number of infected Purkinje cells counted in a one out four series of serial sections of the anterior lobe (5-day-case: *n* = 1592, 6-day-case: *n* = 7458) only a very marginal increase from 115 to 139 infected olivary cells (counted in a one out four series of serial sections) was noted. A similar count of infected olivary neurons of the case where degeneration of the B-zone was described (see **Figure 4**) resulted in only 29 infected olivary cells.

#### Molecular Layer Interneurons and Granule Cells Are Not Infected from Labeled Purkinje Cells

**FIGURE 6:**
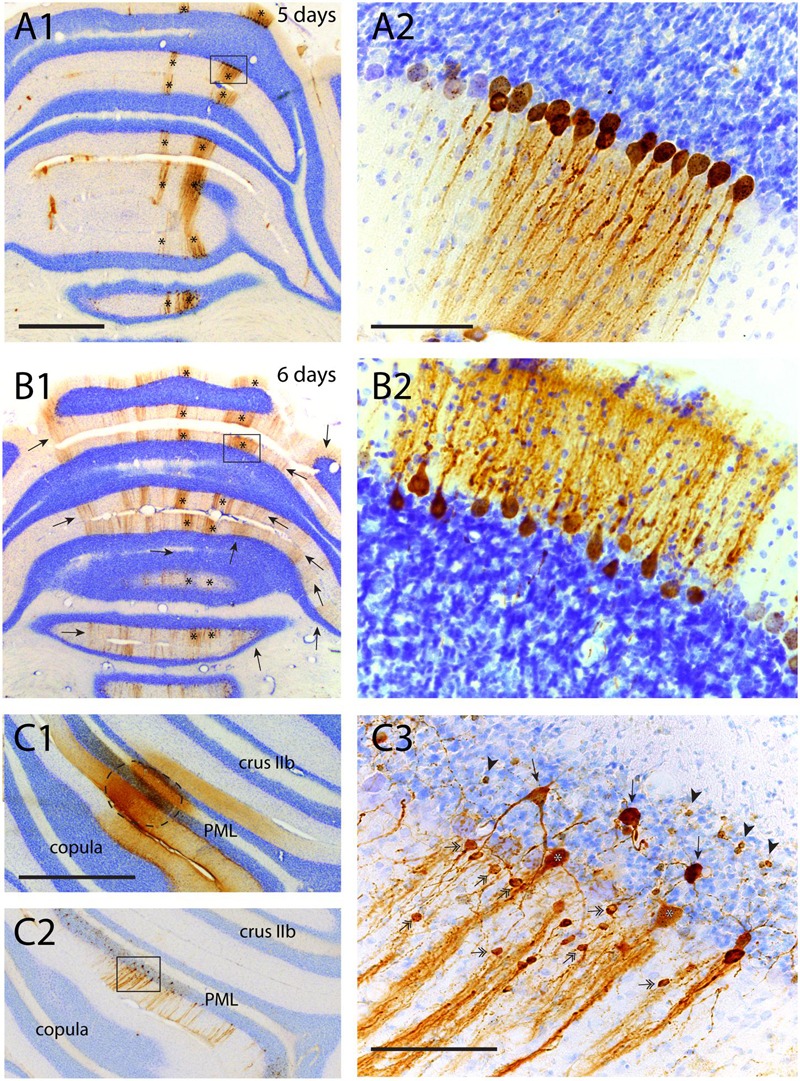
**Failure of transneuronal infection from Purkinje cells to molecular layer interneurons and granule cells. (A1,2)** First appearance of labeled neurons 5 days after injection of anterior tibial muscle. Strips of infected Purkinje cells are seen in the lateral A-zone and within the B-zone of the cerebellar cortex (marked by asterisks). Boxed area in **(A1)** is enlarged in **(A2)**. Within the B-zone, as expected, no other neurons than Purkinje cells are labeled. **(B1,2)** Again A- and B-zone labeling (asterisks) in a similar experiment but now with a survival time of 6 days, resulting in many additional RABV-labeled strips of Purkinje cells. However, careful examination of the B-zone did not show any infected stellate, basket or granule cells. **(C1–3)** Injection site (encircled) of RABV/CTb mixture injected directly in the cerebellar cortex. **(C1)** CTb immunohistochemistry, **(C2)** RABV immunohistochemistry. **(C3)** Detail of boxed area shown in **(C2)**. Note many labeled types of cerebellar cortical interneurons (e.g., stellate/basket cells: double headed arrows; Golgi cells, arrows; granule cells: arrowheads). Scale bars equal 1 mm **(A1–C1,C2)** and 100 μm **(A2,B2,C3)**.

As noted above, RABV injection in hind limb muscles resulted in infection of Purkinje cells that was first noted at a survival time of 5 days in two vermal strips of which the B-zone was the most prominent one (Ruigrok et al., 2008). In the cerebellar cortex no infection of cells other than Purkinje cells was apparent (**Figures 6A1,2**). At a survival time of 6 days additional strips became apparent in the vermis but also in the hemispheres and indicated that enough time had elapsed for the RABV to pass an additional synaptic step (cf. **Figure 1A**). However, at this stage close inspection of the cerebellar cortex of the infected Purkinje cell strips labeled after 5 days (i.e., of the B-zone), did not reveal any subsequent infection of neurons presynaptic to the Purkinje cells (**Figures 6B1,2**). Yet, in the same animal, transfection from layer V pyramidal cells to cerebral cortical interneurons, had occurred in great numbers (not shown, note that infection of the first Purkinje cells occurs at the same time as first infection of layer V pyramidal cells, cf. **Figure 1A**). Even 8 days after injection, at a time when infected Purkinje cells started to degenerate, no labeled interneurons, such as basket or stellate cells were noted in the molecular layer (cf. **Figures 4A1,2**). In addition, in all these cases no evidence of infected granule cells was observed.

Rabies virus-infected molecular layer interneurons, granule cells and also Golgi cells, however, can always be observed when a combination of RABV and CTb was injected directly into the cerebellar cortex (**Figures 6C1–3**) at survival times sufficient for second or third order labeling (Suzuki et al., 2012). This suggests that the transneuronal transfer of the ‘French’ CVS11 strain of RABV from Purkinje cells to their presynaptic element seems to be seriously curbed, despite permissiveness of these cell types for RABV entry from the extracellular space.

#### Nucleo-olivary Neurons Are Not Infected after RABV Injection into the Inferior Olive

**FIGURE 7:**
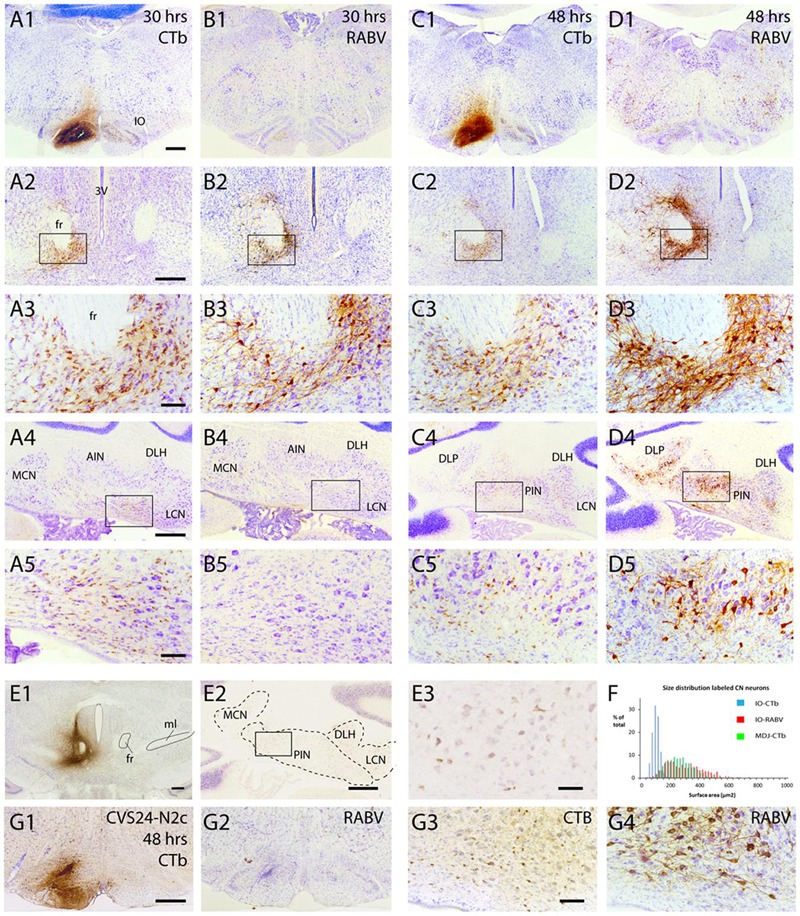
**Failure of axonal RABV uptake and replication by nucleo-olivary neurons. (A1–5)** CTb labeling resulting from injection of RABV/CTb mixture in the inferior olive with a survival time of 30 h. **(A1)** Injection site covers most of the inferior olivary complex without obvious invasion of surrounding reticular formation. **(A2)** Labeling of olivary afferents around the fasciculus retroflexus (fr). **(A3)** Detail of boxed area shown in **(A2)**. **(A4)** Labeling of olivary afferents in the cerebellar nuclei. **(A5)** Detail of boxed area shown in **(A4)**. Note the abundance of small CTb-labeled nucleo-olivary neurons. **(B1–5)** RABV labeling of adjacent sections shown in **(A1–5)**. Note that virtually no RABV labeling is visible in the inferior olive **(B1)**, but that abundant RABV labeling can be observed around the fr **(B2,3)**. However, the cerebellar nuclei are completely devoid of RABV labeling **(B4,5)**. **(C1–5)** Similar to **(A1–5)** but now after a survival of 48 h. CTb labeling is very reminiscent of the situation after 30 h for all figures. **(D1–5)** Same animal as shown in **(C1–5)**, but now depicting adjacent sections immunostained for RABV. RABV labeling in inferior olive is still very poor despite infection of surrounding reticular formation **(D1)**. Labeling around fr has become quite prominent **(D2,3)**. At this time point RABV-infected neurons were prominently visible in the cerebellar nuclei **(D4,5)**. However the size of the RABV-labeled neurons was quite different from the CTb-labeled neurons (cf. **C5,D5**). **(E1–3)** CTb injected around the fr **(E1)** result in retrogradely labeled neurons in the cerebellar nuclei **(E2,3)**, suggesting a nucleo-midbrain-olivary connection (De Zeeuw and Ruigrok, 1994). **(E3)** Enlargement of boxed area shown in **(E2)**. **(F)** Size distribution of cerebellar nuclear neurons retrogradely labeled from three injections centered on the fr area in **(E1**; green bars, *n* = 885 in three cases), compared to the size distribution of RABV-infected neurons (red bars, *n* = 608 in three cases) and CTb-labeled neurons (blue bars, *n* = 1216 in three cases) after RABV/CTb injection into the inferior olive and a survival time of 48–50 h (enabling second-order RABV labeling). Note that the green and red bars are completely overlapping, suggesting that they represent the same populations, whereas the neurons that project to the olive represents a very distinct population of small cells, not overlapping with the green and red distributions. **(G1–4)**, Similar to **(C1,5)** and **(D1,5)** but now depicting results from an olivary injection with the RABV strain CVS24-N2c. **(G1,2)** show the injection site with CTb and RABV immunohistochemistry, respectively. **(G3,4)** show small CTb-labeled nucleo-olivary cells in the cerebellar nuclei **(G3)**, whereas an adjacent section only shows large RABV infected neurons **(G4)**, very similar to the results obtained with the ‘French’ CVS11 strain used in the all other experiments (cf. **C5,D5**). Scale bars represent 500 μm **(A1–D1,A2–D2,A4–D4,E1,2,G1,2)** or 100 μm **(A3–D3,A5–D5,E3,G3,4)**. Abbreviations: 3V, third ventricle; AIN, anterior interposed nucleus; DLH, dorsolateral hump; DLP, dorsolateral protuberance; fr, fasciculus retroflexus; IO, inferior olive; LCN, lateral cerebellar nucleus, LVN, lateral vestibular nucleus; MCN, medial cerebellar nucleus; ml, medial lemniscus; PIN, posterior interposed nucleus.

In another series of experiments, a mixture of RABV and CTb was injected into the inferior olivary complex. Survival times were chosen to allow for either first-order (30 h), first- and second-order (48–50 h), or first-, second-, and third-order labeling (66–68 h). Since CTb does not pass transneuronally, it only labels first-order neurons retrogradely, i.e., neurons that are directly presynaptic to the olivary neurons. Main excitatory afferent systems of the olivary complex are, apart from the spinal cord, several nuclei, centered around the fasciculus retroflexus, at the MDJ (Ruigrok et al., 2015). In addition, a large group of small GABAergic neurons in the cerebellar nuclei targets the inferior olive massively (de Zeeuw et al., 1989; Ruigrok and Voogd, 1990; Fredette and Mugnaini, 1991). The relevant circuitry is shown in **Figure 1B1**. **Figure 7** shows results from two cases with a RABV/CTb injection into the inferior olive but with different survival times. The injection site, as illustrated by CTb immunohistochemistry (**Figures 7A1,C1**), of both cases was mostly confined to the olivary complex with only some light incorporation of the overlying reticular formation. When allowing only first-order labeling (survival time of 30 h), retrograde transport of CTb showed, as expected, prominent labeling of olivary afferents at the MDJ (**Figures 7A2,3**) as well as of many small nucleo-olivary neurons in the cerebellar nuclei (**Figures 7A4,5**). Adjacent sections, processed for RABV immunohistochemistry, showed that already 30 h after injection RABV could no longer be visualized at the site of injection (**Figure 7B1**). Indeed, probably due to the lack of recurrent collaterals (Ruigrok et al., 1990), hardly any olivary neuron within the injection site had been infected. Yet, successful uptake, transport and replication of RABV had taken place as evidenced by profuse infection of neurons at the MDJ (**Figures 7B2,3**), very similar to the location of the CTb-labeled neurons. However, and highly unexpected, no RABV labeling was observed in the cerebellar nuclei (**Figures 7B4,5**). Prolonging the survival time to 48 h resulted in a similar labeling pattern for CTb (**Figures 7C1–5**). RABV labeling, now allowing for a transneuronal step, showed, apart from similar but denser labeling at the MDJ (**Figures 7D2,3**), also abundant labeling in the cerebellar nuclei (**Figures 7D4,5**). However, when comparing CTb and RABV labeling (cf. **Figures 7C5,D5**) it is immediately clear that the RABV-infected cells were much larger than the small CTb-labeled nucleo-olivary neurons. Indeed, cell measurements not only indicate that the average size of CTb- and RABV-labeled neurons was significantly different but also that within the size distribution of the RABV neurons the population of small cells was virtually absent (**Figure 7F**). Moreover, the distribution of RABV-infected soma sizes after 48 h was very similar to that observed after injection of CTb at the MDJ (**Figures 7E1–3,F**). This strongly suggests that the observed RABV infection in the cerebellar nuclei 48 h after injection only represented second order labeling and failed to infect the nucleo-olivary neurons (cf. **Figure 1B2**). Indeed, strong projections of the cerebellar nuclei to the MDJ area surrounding the fasciculus retroflexus have been repeatedly described (Teune et al., 2000; Ruigrok et al., 2015) and form the basis of an excitatory disynaptic pathway from the cerebellar nuclei to the inferior olive (De Zeeuw and Ruigrok, 1994; Ruigrok and Voogd, 1995). It should be stressed that at 48 h no Purkinje cells were infected, which would be expected from a disynaptic route from Purkinje cells to nucleo-olivary neurons (De Zeeuw and Berrebi, 1995; Teune et al., 1998).

**FIGURE 8:**
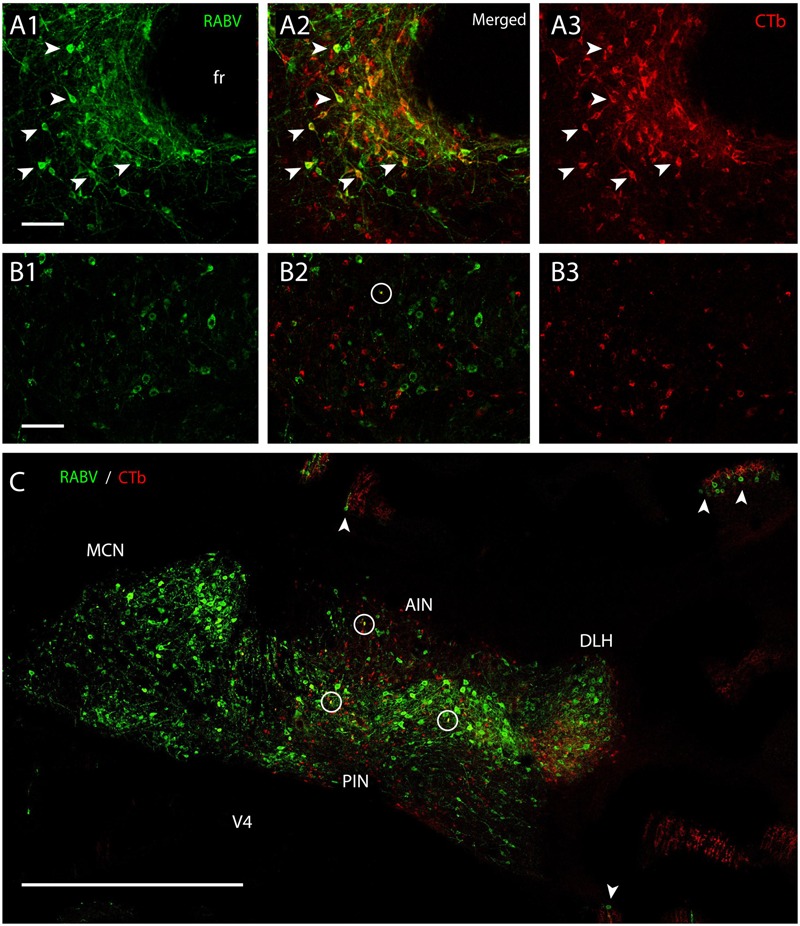
**Separation of CTb- and RABV-labeled neurons after combined injection in the inferior olive. (A1–3)** Fluorescent double labeling of RABV- (green) and CTb- (red) labeled cells around the fasciculus retroflexus (fr). Arrowhead indicated some of the double-labeled neurons (survival time 48 h). **(B1–3)** No double labeling, apart from a single cell (encircled) is seen in the cerebellar nuclei of another injection after an even longer survival time of 66 h. **(C)** Overview of the cerebellar nuclei indicating complete intermingling of RABV-infected (green) and CTb-labeled neurons of the same animal shown in **(B1–3)**. Only a few double-labeled profiles are noted (encircled). Note that in this case RABV infection of Purkinje cells (arrowheads) can also be observed which is also accompanied by anterogradely labeled climbing fibers (red profiles at arrowheads). Scale bars represent 100 μm **(A1–3,B1–3)** and 1 mm **(C)**.

Fluorescent double labeling further strengthened the conclusion that the CTb- and RABV-labeled cells in the cerebellar nuclei seen after a survival time of 48 h represented two distinct populations of neurons (**Figure 8**). Although neurons double-labeled by both CTb and RABV were frequently observed at the MDJ (**Figures 8A1–3**), they were not, or hardly, observed within the cerebellar nuclei even after 68 h (**Figures 8B1–3,C**). Only at this survival time, normally resulting in third-order labeling (Suzuki et al., 2012), the first RABV-infected Purkinje cells became visible, which were usually aligned with the strips of climbing fibers anterogradely labeled with CTb (**Figure 8C**, arrowheads). We conclude that these RABV-infected Purkinje cells represented third-order labeling and resulted from their innervation of second-order RABV neurons that contact the MDJ neurons projecting to the inferior olive (cf. **Figure 1B2**).

The apparent failure of RABV uptake within the time allotted for normal first-order infection was also seen when another strain of RABV (i.e., CVS 24-N2c) was injected into the inferior olive (*n* = 1, ST = 48 h **Figures 7G1–4**). This suggests that the failure of proper uptake was not only restricted to the specific type of RABV which was customarily used in our experiments (i.e., the ‘French’ CVS11).

As virtually no neurons were labeled within the inferior olive itself after time intervals of 30 and 48 h (**Figures 7B1,D1**), we suggest that although the virus, as expected, can directly penetrate the axon terminals of the excitatory neurons that are located at the MDJ, they apparently cannot invade the GABAergic nucleo-olivary terminals in a similar way.

#### Small Neurons in the Cerebellar Nuclei Can Be Infected Transneuronally

**FIGURE 9:**
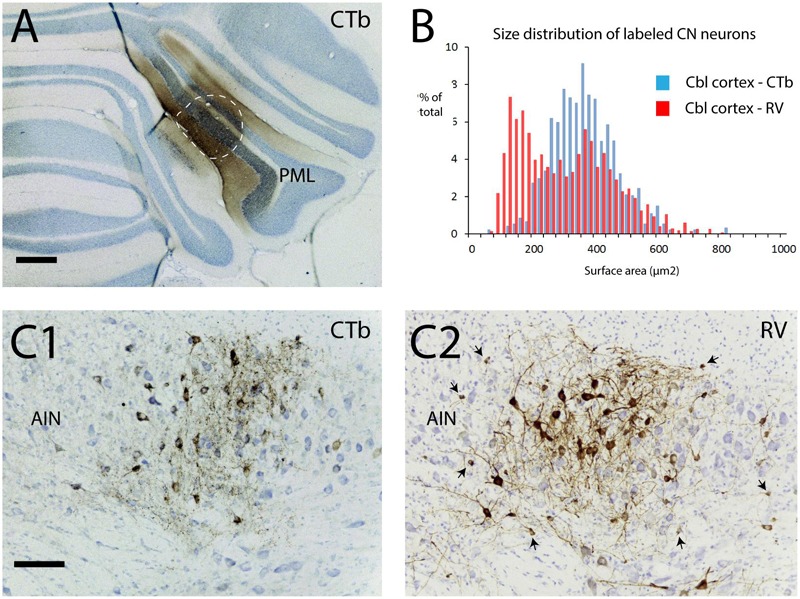
**Second-order RABV infection of a population of small cells in the cerebellar nuclei after cerebellar cortex injection of RABV/CTb mixture.** This experiment shows that the population of small cells can be transneuronally infected by RABV. **(A)** Injection of RABV/CTb mixture in PML (encircled, section immunostained for CTb). **(B)** Size distribution of CTb-labeled and RABV-infected neurons in the cerebellar nuclei after a survival time of 48 h (allowing for second-order RABV infection). Note that the RABV-infected cells also represent a sizeable contingent of small cells, which, at least partly, overlaps with the size distribution of neurons that project to the inferior olive (cf. **Figure 7F**, blue bars). **(C1)** Example of CTb immunostaining in the AIN. Only large labeled neuronal profiles are seen. **(C2)** Adjacent section immunostained for RABV. Apart from large profiles now also many small profiles can be observed (arrows). As the small profiles were absent in **(C1)**, they are thought to be the result of transneuronal infection. Scale bars represent 500 μm in **(A)** and 100 μm in **(C1,2)**.

In another set of experiments we show that small neurons of the cerebellar nuclei, within the same size category as the nucleo-olivary fibers, can become infected using a transneuronal route. This time, the RABV-CTb mixture was injected into the PML of the cerebellar cortex (**Figure 9A**). After 48 h, thus allowing for second-order labeling, many neurons were RABV-infected within precerebellar nuclei such as the inferior olive, the lateral reticular nucleus, and the reticular and basal nuclei of the pons (Suzuki et al., 2012). As expected, these areas also contained CTb-labeled neurons indicating that the RABV labeling represented first-order labeling. Prominent labeling of both RABV and CTb neurons was also noted within the cerebellar nuclei, indicating nucleocortical connections to the PML (**Figures 9C1,2**) (Houck and Person, 2014; Gao et al., 2016). However, RABV infection of CN neurons could also be accomplished by second-order labeling of, e.g., PML-IO-CN, PML-pontine-CN or PML-CN-CN retrograde routes. Yet, analysis of the size distribution of CTb- and RABV-labeled neurons learned that the RABV population contained a sizable contingent of small neurons, whereas the CTb-labeled population did not (**Figure 9B**), thereby indicating that the small cells do not project to the cortex (Ankri et al., 2015). As small neurons also do not project to precerebellar nuclei other than the inferior olive (Teune et al., 1995; Ruigrok and Teune, 2014), the population of small cells could only be infected either as second-order step through the inferior olive or through local collateral connections to the first-order labeled nucleocortical neurons (cf. **Figure 1B3**) (Uusisaari and De Schutter, 2011). Although the precise route cannot be ascertained in this type of experiment, we can conclude that failure of uptake and transport of RABV by olivary injection of RABV does not imply that the population of small nucleo-olivary cells is completely resistant to RABV infection.

## Discussion

The ‘French’ rabies strain CVS11 is frequently used to examine circuitry within the brain (e.g., see Ugolini, 1995, 2008; Morcuende et al., 2002; Moschovakis et al., 2004; Suzuki et al., 2012). Similar results have been reported for the related strain N2c of CVS24 (Kelly and Strick, 2000). These strains have been presented as extremely powerful tools for examining synaptically connected chains of neuronal elements (Ugolini, 2010, 2011). However, although we are also impressed with the potential of this tracer for neuroanatomical research, in the present set of experiments we came upon some results that, in our opinion, need to be considered carefully in the interpretation of data. In particular, we claim that (1) gliosis is generally seen early in heavily infected areas; (2) some types of neurons may succumb to RABV infection within several days after infection; (3) not all types of neurons are readily infected.

### Invasion of Microglia

Successful infection from the viral point of view is ascertained when, after initial infection by, e.g., a bite by a rabid dog or bat, the virus can reach the central nervous system by retrograde transport to motoneurons, subsequent replication and successful transneuronal propagation through the nervous system until it can infect the salivary glands. At that time the virus can be propagated to other animals for a new cycle. During its journey through the central nervous system it is essential that the virus is not attacked by the host’s defense mechanisms. Indeed, RABV-infected neurons can successfully counteract invasion of T-cells and minimize inflammation (for review, see Lafon, 2011). Simultaneously, successful RABV infection should preserve cellular integrity and prevent apoptosis of the cell despite the fact that the infected neuronal soma and dendrites are completely filled with viral material (Ugolini, 2010, 2011; Lafon, 2011).

In our material, we note profound gliosis of heavily infected areas. A gliotic response already can be noted 48 h after injection within areas that show first-order labeling, indicating that gliosis may start within hours after replication of the virus within the infected neurons. Occasionally, accumulations of microglia could be observed directly around an RABV-infected neuron. Such a neuron usually displayed an affected morphology (beaded dendrites, vacuoles, and irregular somatic morphology). However, in the same region many other infected neurons did not show such accumulations of glia and displayed a normal general morphology. As far as we know, differences in glial response and affected morphology within the same animal has not been reported before as a potential characteristic trait of RABV infection (i.e., of the ‘French’ CVS11 strain; Ugolini, 2010, 2011). Yet, it seems likely that a profound proliferation of microglia will have its effects on the physiology of the infected neurons.

### Degeneration of RABV-Infected Neurons

An important advantage of using RABV as an transneuronal tracer would be that it does not affect the appearance and functioning of infected neurons for a considerable time. In rats, motoneurons have been reported to remain apparently unaffected for at least 4 days and, as the infected animals remain asymptomatic during that period, potentially for up to a week after injection (Ugolini, 1995, 2010; Tang et al., 1999; Kelly and Strick, 2000; Morcuende et al., 2002). Yet, after careful examination of our material we clearly see examples of cells that were degenerated within that time frame. Deposits of reaction product assumed to be necrotic somata and that were located within the motoneuron pool containing ‘normal’ appearing RABV-infected motoneurons clearly suggest that accumulation of virus caused the destruction of the cell by a process that induces cellular lysis and seems quite dissimilar from apoptosis. It is important to note that no signs were observed that the deposits of reaction product could serve as a new source of infection as has been reported for, e.g., Herpes Simplex virus (Ugolini, 2010). As these specific deposits were already observed at 4 days post-injection and infection of motoneurons after RABV injection into muscles in rat has been reported to take 2 or 3 days (Tang et al., 1999; Graf et al., 2002; Morcuende et al., 2002) we conclude that spinal motoneurons innervating limb muscles may be especially vulnerable to RABV infection and may degenerate 1 or 2 days after infection. Moreover, as an *in vitro* study has demonstrated that isolated RABV-infected embryonic rat motoneurons can withstand apoptosis for at least 7 days in culture (Guigoni and Coulon, 2002), this would imply that the observed lytic process of the motoneurons in our material is induced by a different, i.e., non-apoptotic, mechanism requiring the intact system. It is remarkable that a similar lytic effect on motoneurons has not been reported in other experimental settings. However, it should be noted that so far CVS 11 muscle injections in rat or guinea pig were confined to the bulbospongiosus muscle, or to oculomotor and facial muscles (Tang et al., 1999; Graf et al., 2002; Morcuende et al., 2002). It could well be that motoneurons that innervate skeletal muscles may be more susceptible to RABV infection and subsequent lysis than, e.g., oculomotor neurons and the neurons of Onuf’s nucleus. In this respect it may be noted that some other disease types, such as amyotrophic lateral sclerosis, also seem to affect these neurons at a much later stage (Mannen et al., 1977; McLoon et al., 2014).

Rabies virus-infected Purkinje cells also were affected in such a way that degeneration occurred within several days. We, and others, have shown that the earliest infected Purkinje cells after RABV muscle injections were observed after 5 days (Tang et al., 1999; Morcuende et al., 2002; Ruigrok et al., 2008). Purkinje cells have also been reported to be more vulnerable to, e.g., alcohol exposure or ischemia than many other neuronal types (Welsh et al., 2002; Lee et al., 2008) and therefore may also succumb earlier than other cell types to RABV infection. In this respect, as all Purkinje cell are calbindin-positive, it should be noted that RABV infection has also been suggested to specifically affect calbindin-positive cells. Downregulation of calbindin expression in cerebral cortex and striatum of mice intramuscularly injected with the ‘fixed’ CVS RABV has been reported at the onset of clinical signs after 7 days (Torres-Fernandez et al., 2005). However, our results show that RABV-infected Purkinje cells in the vermal region still demonstrate the same level of calbindin expression as surrounding non-RABV-infected cells (cf. **Figures 4A1,B1**). Calbindin expression only disappears when the cells themselves have degenerated. Furthermore, it should be noted that the degenerated Purkinje cells did not resemble the lytic process observed for the infected motoneurons, suggesting that a different, possibly innate, process of cell destruction has taken place.

As in rat both spinal motoneurons and Purkinje cells may be liable to degeneration within 1 or 3 days after infection with the ‘French’ CVS 11 strain of RABV, in our view it seems too optimistic to state that rabies infection and replication does not cause cell damage within a week (Ugolini, 2010, 2011).

### Poor or Failure of RABV Infection

Even more remarkable than the early degeneration of infected neurons was our observation that some neuronal types that were expected to become infected as they were synaptically connected to infected neurons did not, or at a much reduced level, actually became infected. Initially, this was observed for labeling of inferior olivary neurons after muscle injections. As most olivocerebellar axons collateralize to the cerebellar nuclei in an orderly organizational fashion (van der Want et al., 1989; Sugihara et al., 1999; Ruigrok and Voogd, 2000; Pijpers et al., 2005) that reflects the olivo-cortico-nuclear modular organization of the cerebellar cortex (Ruigrok, 2011; Ruigrok et al., 2015), it was expected that, simultaneously with Purkinje cell labeling within the cerebellar cortex, labeling of olivary neurons would occur. However, despite the infection of prominent strips of Purkinje cells, classified as belonging to the B-zone (Voogd and Ruigrok, 2004; Ruigrok, 2011), only a relatively small number of olivary neurons, mainly localized in the dfDAO were infected in the same animal. Poor infection of olivary neurons might be related to the observation that climbing fiber collaterals to the cerebellar nuclei are of a fine caliber category and tend to terminate on distal dendrites (van der Want and Voogd, 1987; Ruigrok and Voogd, 2000). However, even at survival times that allow for labeling of an additional transneuronal step, no substantial increase in either number or location of infected olivary areas was observed (Ruigrok et al., 2008). It should be noted that at these time points olivary neurons of the dfDAO not only had been exposed to transfection from infected neurons of the LVN (that serves as target of the Purkinje cells of the B-zone) but also to transfection from the infected B-zone Purkinje cells. Yet, despite the unparalleled strength of the climbing fiber to Purkinje cell synaptic connection, only a minority of olivary cells that were eligible for infection were in fact RABV-positive. It seems likely that the synaptic contacts of olivary axons are instrumental in their poor infection results. When RABV is injected directly into the cerebellar cortex, localization and density of labeled olivary neurons are completely consistent with results of retrograde transport of CTb (Suzuki et al., 2012).

The problem of further transneuronal transport from infected Purkinje neurons was also noted by the failure to label local interneurons that are presynaptic to them. Basket neurons, which form strong synaptic contacts with soma, axon hillock, and initial segment of Purkinje cells, but also stellate cells that contact the dendritic tree, were not transfected from the infected Purkinje cells of the B-zone, despite the fact that there was ample time to allow additional transfer. Likewise, granule cells were not observed at any time point. Even in one rat, where the hind limb muscle injection was made 8 days before and had resulted in degeneration of Purkinje cells in the B-zone of the cerebellar cortex, no cortical interneurons were found anywhere in the cerebellar cortex, including the strip of cortex containing the degenerated Purkinje cells. As in the case of olivary cells it would seem that the synaptic specializations between Purkinje cells and their presynaptic elements are the limiting factor and are instrumental in the failure of viral transfer. Injection of RABV directly in the cerebellar cortex resulted, apart from labeling of Purkinje cells, in selective infection of both types of molecular interneurons as well as of granule cells and Golgi cells in the granular layer. This suggests that the capacity of neurons to become infected by transneuronal RABV transfer may be different from RABV uptake from the extracellular space by either their local axonal terminals or soma-dendritic membrane.

It should be noted that our results contrast a study by Kelly and Strick (2004) where the ‘American’ CVS 11 strain was injected in M1 of the monkey cortex and reportedly resulted in fourth-order labeling of granule cells. This study does not explicitly mention whether or not molecular layer interneurons were labeled. The difference might be attributed to the different host (i.e., monkey vs. rat) and/or to the ‘American’ CVS 11 strain used in Kelly and Strick’s study as this has been reported to be somewhat different from the ‘French’ CVS 11 strain (Ugolini, 2010).

The suggestion that viral transneuronal transport from postsynaptic element to presynaptic element might be essentially different from viral uptake from the extracellular space to presynaptic elements was also strengthened by the apparent failure of nucleo-olivary fibers to become infected after RABV injection into the inferior olive. At survival times that were sufficient for first- (30 h) and second-order (48–50 h) infection, the category of small nucleo-olivary neurons were missing in the size distribution of RABV-labeled neurons in the cerebellar nuclei. Also, no Purkinje cells were infected at the latter time point despite the fact that nucleo-olivary neurons are also targeted by Purkinje cells (De Zeeuw and Berrebi, 1995; Teune et al., 1998; Najac and Raman, 2015). This effect was similar for both the ‘French’ CVS 11 as for the CVS 24 N2c strain.

Interestingly, however, a prominent population of small cells in the same size category as nucleo-olivary neurons became infected using another route. We conclude that in this case transneuronal transfer either by local collaterals of nucleo-olivary neurons or by first order infection of olivary neurons and subsequent transneuronal transfer did enable infection of nucleo-olivary neurons. So, in some situations RABV infection seems to be curbed by synaptic specializations (e.g., molecular layer interneuron to Purkinje cell) but is allowed from the extracellular space, whereas in other situations (e.g., nucleo-olivary terminal to inferior olivary cell) the virus cannot reach the axon from a position extracellular of the axon terminals but may succeed in transneuronal transfer by way of the synaptic contact.

Importantly, we describe several cases where expected labeling did not, or only reluctantly, occur. This contrasts the general notion described in literature that in all models studied so far, RABV did not fail to label neurons that were presynaptic to either injection site or RABV-infected neurons (Ugolini, 1995, 2010, 2011; Kelly and Strick, 2000, 2003).

## Conclusion

The use of unmodified ‘fixed’ strains of RABV as a transneuronal tracer to visualize synaptically connected chains of neurons remains a very potent tool, especially when examination of second-, third-, or higher-order structures is at stake. However, we strongly stress that researchers need to be careful and examine for every connection they want to study if RABV is indeed readily taken up and transferred as expected within the allotted time interval. In addition, fate of infected neurons should be carefully monitored over time and may result earlier in physiological or behavioral effects than formerly believed. Based on the wealth of data provided in the literature indicating the contrary, we believe that the anomalies noted here may be exceptions to the generally wide applicability of the RABV tracing technique. Nevertheless, we strongly advocate that researchers using this powerful technique remain wary of its potential caveats. Furthermore, we note that the observed abnormalities (early degeneration of some cell types, failure of uptake or transneuronal transfer) deserve further study in order to improve our understanding of RABV infection processes within the brain.

Finally, we like to stress that from the present study no assertions can be inferred with respect to the genetically engineered types of RABV that are frequently used in mice. However, as a recent report shows that engineered versions of CVS-N2c have prominently different transport qualities compared to engineered versions of the more frequently used SAD-B19 strain of RABV (Reardon et al., 2016) it would seem possible that similar caveats as described here for the unmodified RABV may also be present for engineered versions of RABV.

## Author Contributions

TR and PC designed and executed the tracer experiments; TR, SvT, and PC prepared, analyzed, and interpreted the data; TR, SvT, and PC wrote the manuscript.

## Conflict of Interest Statement

The authors declare that the research was conducted in the absence of any commercial or financial relationships that could be construed as a potential conflict of interest.
